# Procedural complication and long term outcomes after alcohol septal ablation in patients with obstructive hypertrophic cardiomyopathy: data from China

**DOI:** 10.1038/s41598-017-10144-0

**Published:** 2017-08-25

**Authors:** Shuo-yan An, Yin-jian Yang, Fei Hang, Zhi-min Wang, Chao-mei Fan

**Affiliations:** 1 Key Laboratory of Clinical Trial Research in Cardiovascular Drugs, Ministry of Health, Fuwai Hospital, National Center for Cardiovascular Diseases, Chinese Academy of Medical Sciences and Peking Union Medical College, Beijing, China; 2 Department of Ultrasound, Fuwai Hospital, National Center for Cardiovascular Diseases, Chinese Academy of Medical Sciences and Peking Union Medical College, Beijing, China

## Abstract

Data on procedural complications and long term survival after alcohol septal ablation (ASA) in Chinese patients with obstructive hypertrophic cardiomyopathy (HOCM) are lacking. We aimed to investigate long-term survival of HOCM patients after ASA and compared to the non-obstructive hypertrophic cardiomyopathy(NOHCM). A total of 233 patients with HOCM and a peak pressure gradient of ≥50 mm Hg at rest or with provocation were consecutively enrolled from Fuwai Hospital in China between 2000 and 2012. Another 297 patients without left ventricular outflow tract obstruction were regarded as control group. Periprocedural mortality of ASA were low (0.89%). Periprocedural lethal ventricular arrhythmia occurred in 9 patients (4.0%). Alcohol volume (RR 1.44, 95% CI: 1.03–2.03, P = 0.034) and age ≤40 years old (RR 4.63, 95% CI: 1.07–20.0, P = 0.040) were independent predictors for periprocedural lethal ventricular arrhythmia. The 10- year overall survival was 94.6% in the ASA group, similar with 92.9% in the NOHCM group (P = 0.930). In conclusion, periprocedural mortality and complications were rare in ASA. Long term survival after ASA were satisfactory and comparable to NOHCM. Patients under 40 years old should be more cautious undergoing ASA, for these patients were more likely to endure lethal ventricular arrhythmia during periprocedural period of ASA.

## Introduction

Hypertrophic cardiomyopathy (HCM) is the most common autosomal dominant heart disease and the leading cause for sudden death in adolescents^1^. In experienced centers, selective injection of alcohol into a septal perforator artery to create a localized septal scar has outcomes similar to surgery in terms of gradient reduction, symptom improvement and exercise capacity, but has the higher incidence of vnetricular arrythmia^2, 3^. In this study, we aimed to investigate long-term survival after alcohol septal ablation (ASA) in the largest Chinese cohort with obstructive hypertrophic cardiomyopathy (HOCM) and compared to the non-obstructive hypertrophic cardiomyopathy (NOHCM).

## Results

### Baseline characteristics

According to the inclusive and exclusive criteria, 530 patients were consecutively enrolled in this study, with 233 patients (43.96%) in the ASA group, and 297 patients (59.04%) in the control group. Patients in the ASA group were older than the NOHCM groups (48.7 ± 9.8 vs 46.2 ± 13.6, P = 0.018). Patients in the ASA groups had more severe symptoms than patients in NOHCM group. More patients in the NOHCM group had atrial fibrillation (19.7% vs 8.6%, P < 0.001), while concomitant of diabetes mellitus, stroke or transient ischemic attack (TIA), coronary artery disease and hypertension had no statistical significance between the two groups (Table 1).

### Procedural data

Mean alcohol volume was 2.5 (1.6) ml during the ASA procedure. Of the 233 patients who underwent ASA, 9 (3.86%) failed the ASA procedure, 2 due to hypotension after contrast agent injected, 1 for cardiac tamponade, and the other 6 didn’t reach the criteria of 50% reduction of LVOTPG from baseline. These patients were excluded from the following analysis. LVOTPG reduced from 93.5 (38.2) mm Hg at baseline to 20.0 (30.0) mmHg (P < 0.001) immediately post-ASA. Residual LVOTPG was 24 (32) mm Hg 3 months after the invasive procedure. Additional septal reduction therapy was required in 16 patients (7.1%) after ASA for high residual pressure gradient or severe symptoms, 12 (5.4%) had myocardial myectomy and 4 (1.8%) had ASA.

### 30-day periprocedural complications

The overall rate of periprocedural mortality was very low in the ASA group, with 2 patients (0.89%) died during the periprocedural period, 1 for retroperitoneal hemorrhage, the other for recurrent ventricular fibrillation. Besides, there were 2 (0.89%) retroperitoneal hematoma and 2 (0.89%) cardiac tamponade, all treated successfully. During the periprocedural periods, 9 patients (4.0%) in the ASA group experienced sustained ventricular tachycardia or ventricular fibrillation (sVT/VF), one patient died (0.44%) after recurrent VF and the other 8 (3.56%) were successfully resuscitated. The multivariate logistic regression analysis indicated that the alcohol volume (RR 1.44, 95% CI: 1.03–2.03, P = 0.034) and age ≤40 years old (RR 4.63, 95% CI: 1.07–20.0, P = 0.040) were significantly associated with higher risk for procedural sVT/VF in the ASA group. Only 1 patient (0.44%) developed permanent atrio-ventricular block and received permanent pacemaker (PPM) implantation.

### Follow-up profiles

In the 521 patients, follow-up was completed in 487 patients (93.3%), and 35 patients (6.70%) were lost to follow-up due to an incorrect or changed address and telephone number in their medical records. Twenty-five (71.4%) of the patients whose recent clinical status could not be ascertained had follow-up period of over 3 years. Follow-up period in the ASA and control groups were 6.03 ± 3.25, and 6.07 ± 4.47 years, respectively (P = 0.911).

### Mortality

**Figure 1:**
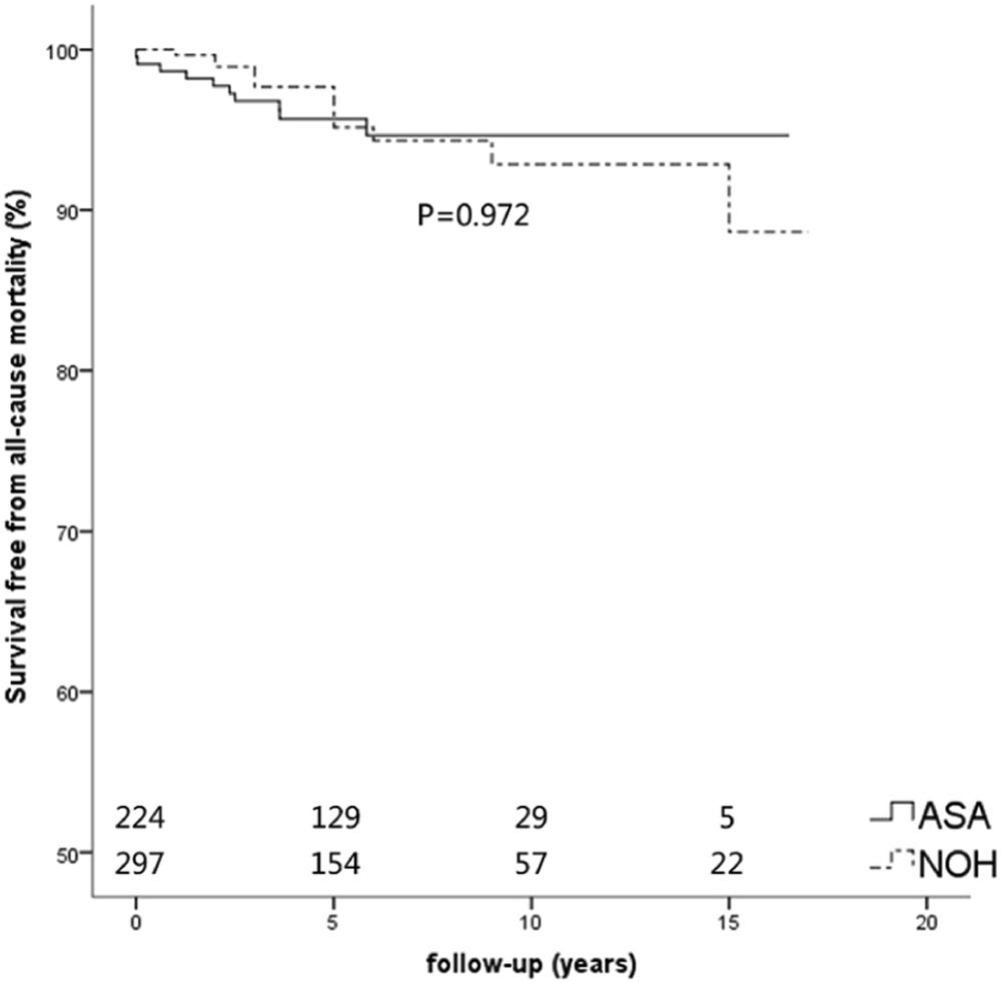
Kaplan-Meier curves of survival free from all-cause mortality. Survival rate in the ASA, and NOHCM group had no statistical significance (P = 0.972). ASA, alcohol septal ablation; NOHCM, non-obstructive hypertrophic cardiomyopathy.

**Figure 2:**
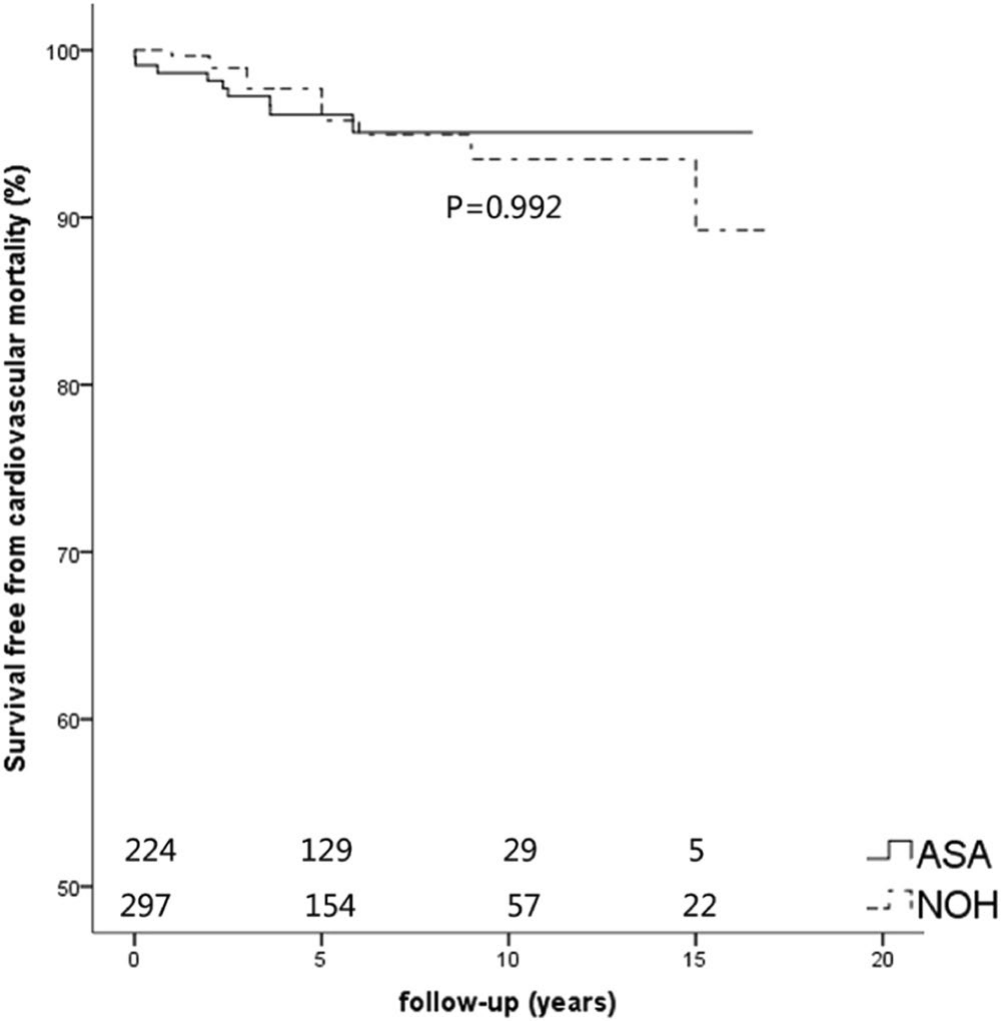
Kaplan-Meier curves of survival free from cardiovascular mortality. Survival rate in the ASA, and NOHCM group had no statistical significance (P = 0.992). ASA, alcohol septal ablation; NOHCM, non-obstructive hypertrophic cardiomyopathy.

**Figure 3:**
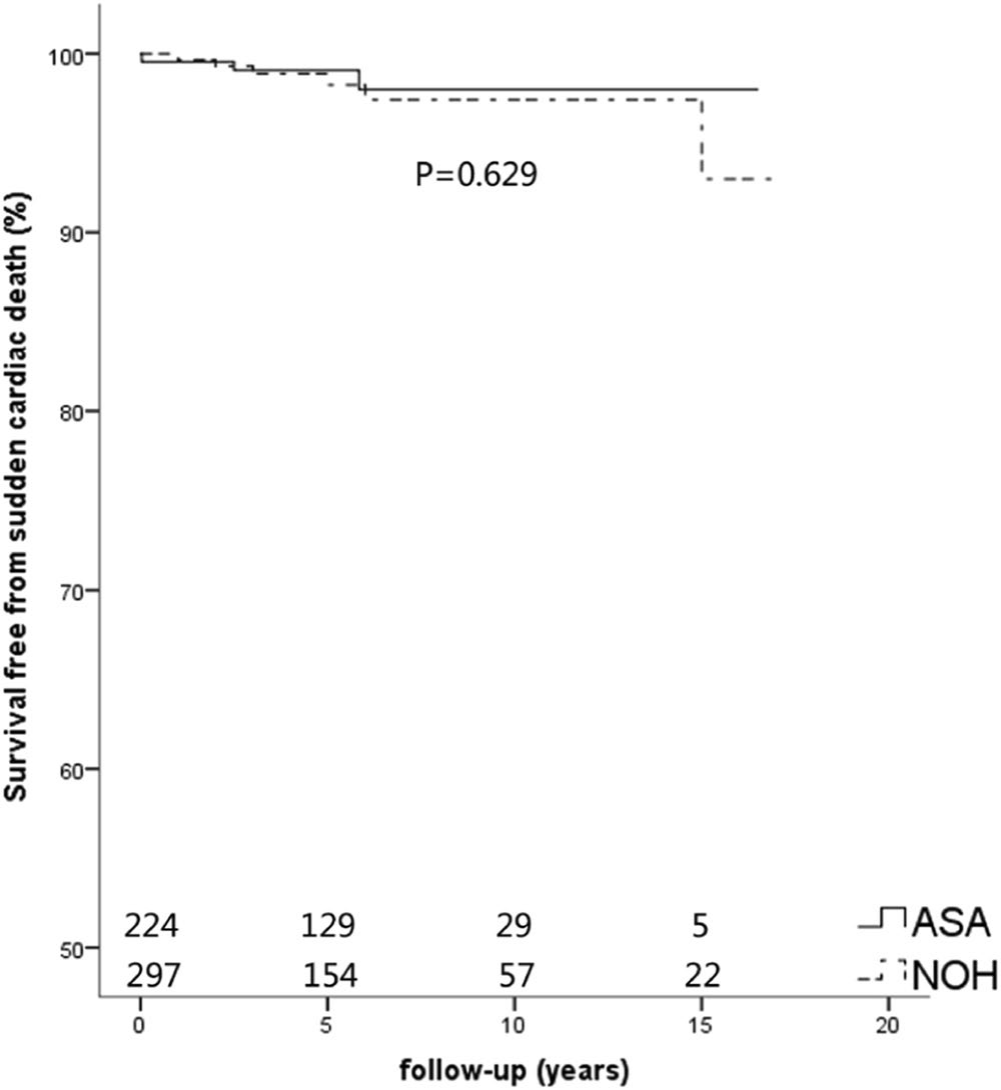
Kaplan-Meier curves of survival free from sudden cardiac death. Survival rate in the ASA, and NOHCM group had no statistical significance (P = 0.629). ASA, alcohol septal ablation; NOHCM, non-obstructive hypertrophic cardiomyopathy.

In the ASA group, 10 patients (4.5%) died during the follow-up period, 9 (4.0%) cardiovascular death (1 for procedure-related complication, 2 for heart failure, 3 for stroke and 3 for SCD), and 1 (0.44%) for malignant tumor. In the NOHCM group, 13 patients (4.4%) died (5 for heart failure, 1 for stroke and 6 for SCD and 1 for unknown reasons). Kaplan-Meier curves of survival free from all cause mortality, cardiovascular mortality and SCD were shown in Figs 1, 2 and 3, respectively. Survival free from all-cause mortality (P = 0.928), cardiovascular mortality (P = 0.870) and SCD (P = 0.641) between the two groups had no statistical significance. The 10- year survival free from all cause mortality was 94.7% in the ASA group and 92.9% in the NOHCM group. In the univariate Cox regression analysis, intraventricular septal thickness (HR 1.138, 95% CI:1.001–1.294, P = 0.049) and residual LVOT pressure gradient (HR 1.032, 95% CI:1.013–1.052, P = 0.001) were associated with all-cause mortality, but in multivariate COX regression analysis only residual LVOT pressure gradient was an independent predictor for all cause mortality (HR 1.037, 95% CI: 1.014–1.061, P = 0.002).

### Age-specific analysis

After dividing ASA population into two groups according to the age, 59 patients (26.3%) were ≤40 years old and 165 patients (73.7%) were older than 40 years old. During the periprocedural periods, 6 patents in the younger group and 3 in the older group experienced sVT/VF (10.2% vs 1.8%, P = 0.011). In the multivariate logistic regression analysis, age ≤40 years old (RR 4.63, 95% CI: 1.07–20.0, P = 0.040) and alcohol volume (RR 1.44, 95% CI: 1.03–2.03, P = 0.034) were independent predictors for periprocedural sVT/VF after ASA. In the long-term prognosis, survival free from all-cause mortality (P = 0.764), cardiovascular mortality (P = 0.611) and SCD (P = 0.778) between the two age-specific groups had no statistical significance.

## Discussion

In this study, we described the complications and long-term outcome of ASA and compared the prognosis with NOHCM in the largest HCM cohort from China. After treated according to the current guideline, these patients showed similar long- term outcome with NOHCM in all cause mortality, cardiovascular mortality and SCD.

After introduction, the efficiency and potential hazard of ASA in the management of symptomatic HOCM patients had been in need of clarity. Ventricular arrhythmia was the main concern of periprocedural and long-term complication after ASA as the myocardial scar might induce electrophysiological disorders^4, 5^.

Occurrence of periprocedural lethal ventricular arrhythmia varied in different studies from 0.8% to 7.4%^5–11^. In our study, the lethal ventricular arrhythmia occurred in 4.0% of the total ASA population. In the multivariate logistic regression analysis, higher alcohol volume and age ≤40 years old were independent predictors for periprocedural lethal ventricular arrhythmia. So the huge variation of sVT/VF among previous studies may attributed to different age and different alcohol volume. The effect of alcohol dosage has been confirmed by several studies earlier^9, 12^. More alcohol used during ASA procedure led to lager infarct size, which induce lethal ventricular arrhythmia^9^. However, effect of age on periprocedural ventricular arrhythmia after ASA has been scarcely discussed. Leonardi *et al*.^10^ compared 360 HCM patients undergoing ASA in 3 age categories ( < 45, 45 to 64, and >65 years). sVT/VF attacked only 0.8% of the whole population without statistical significance among the three age categories. M Liebregts *et al*.^11^ compared the short-term effect after ASA between patients ≤55 years old and patients >55 years old, and 8% patients ≤55 years old experienced adverse arrhythmic events, similar with 7% patients >55 years old (P = 1.0). In the present study, incidence of sVT/VF in patients ≤40 years old was 10.2%, much higher than 1.8% in patients >40 years old (P = 0.011). Different result may be explained by the different cutoff age in these studies. In the 2011 AHA guideline for the management of HCM, ASA was discouraged in adults less than 40 years of age if myectomy is available. This statement was C level of evidence, which meant only consensus opinion of expertise. In the present study, part of the patients received ASA before the guideline published, so patient younger than 40 years old had ASA procedure. Our large-scale retrospective study provided proof for the statement in the guideline that patients younger than 40 years old were less encouraged to undergo ASA for susceptibility of periprocedural sVT/VF.

Besides periprocedural effects, several large-scale studies had discussed the long-term effect of ASA^5, 7, 8, 13–19^ and revealed benign prognosis after this invasive procedure. In the present study, we presented the long-term outcome after ASA in the largest Chinese cohort. Similar with previous studies, the 10- year survival free from all-cause mortality was satisfactory and equivalent to NOHCM. However, whether the alcohol induced myocardial infarction heightened SCD rate after ASA was still in debate. Earlier studies by ten Cate FJ *et al*.^9^ and Noseworthy PA *et al*.^4^ reported higher SCD rate after ASA than myectomy. But subsequent studies^5, 18, 20^ and meta analyses^21–23^ showed that SCD after ASA was uncommon and similar to myectomy. In our study, annual incidence of SCD was 0.3% after ASA, equivalent to NOHCM. Since more focus on younger patients, two previous study analyzed the age-specific long-term prognosis. Leonardi *et al*.^10^ compared the outcomes of HOCM patients undergoing ASA and revealed similar one-year mortality in 3 age categories (<45, 4 5 to 64, and >65 years old), but SCD rate was not compared. In the study by M Liebregts^11^, they compared all-cause mortality after ASA in patients ≤55 years old with age- matched NOHCM, and no statistical significance was found. Patients >55 years old also had no statistical significance compared with age- matched NOHCM. But all-cause mortality and SCD rate after ASA between patients ≤55 years old and >55 years old were not compared. In our study, we compared the all-cause mortality, cardiovascular mortality and SCD rate between two age groups and there was no statistical significance.

Atrioventricular block was a common complication after ASA. In early studies, the occurrence of new PPM implantation after ASA was as high as 25.9%^17^, more than myectomy^7, 18^. However, in the study by Steggerda RC, the incidence of new PPM in the ASA group was 7%, similar with myectomy group^5^. In our study, new PPM was applied in 0.44% of ASA population, far less than studies reported before. This could be explained by the younger age of ASA population in our study. In the study by J Veselka^24^, age was a predictor for atrioventricular block with borderline statistical significance (P = 0.05). In the study by Liebregts M^11^ and RA Leonardi^10^, after dividing ASA patients into different age group, younger patients were with lower incidence of new PPM implantation than older patients. In early studies and meta-analyses, mean age of ASA population was more than 50 years old^8, 19, 23^ while in our study, mean age of ASA population was less than 50 years old, so the need for PPM was lower than reported before. Besides the age, alcohol volume plays a role in the atrioventricular block^19, 25^. In earlier studies, high dose of alcohol was used during ASA to achieve lower pressure gradients, but studies showed that the advantage of lower pressure gradients was balanced by higher incidence of atrioventricular block^3, 19^. In the present study, mean alcohol volume was 2.5 ml, less than some earlier studies^13, 26–28^. Young age and low alcohol volume together made the lower PPM implantation in our study.

Prediction of post-ASA outcome is challenging because of the marked heterogeneity of the treated HCM cohort. In our study, residual LVOT gradient (HR 1.037, 95% CI: 1.014–1.061, P = 0.002) is an independent predictor for all-cause mortality in the ASA population. This relation between residual LVOT gradient and outcome has been reported previously^18, 19^. It is essential to find more accurate septal perforator artery in the process to use less alcohol and get more reduction in LVOT gradients.

This study had some clinical implications. Periprocedural mortality was uncommon after ASA. Survival free from all-cause mortality, cardiovascular mortality and SCD were similar between ASA and NOHCM patients. However, patients under 40 years old were less encouraged to have ASA, for these patients were more likely to endure sVT/VF during periprocedural period of ASA, but long-term SCD rate between different age groups had no statistical significance. Higher residual LVOT gradients might predict worse prognosis, so precise septal perforator artery was vital in the ASA procedure.

There are some limitations in our study. First, there are some inevitable bias as this is a nonrandomized, observational study, but we have try our best to minimize the bias by consecutively enrolled the patients and complete the follow-up. Second, due to the economic restraints, most patients with high risk of SCD didn’t receive ICD implantation as recommended, so the aborted SCD was not calculated as the endpoint.

## Conclusion

Long-term prognosis after ASA is satisfactory. The all-cause mortality, cardiovascular mortality and SCD rate after ASA is similar to that of patients with NOHCM. Patients younger than 40 years old are discouraged to have ASA for the high incidence of ventricular arrhythmia during periprocedural period.

## Methods

### Study population

This study population consisted of adult patients (≥18 years of age at initial presentation) with HCM underwent ASA in Fuwai Hospital between 2000 and 2012. HCM patients without left ventricular outflow tract obstruction (LVOTO) after provocation were considered to be NOHCM and regarded as the control group. The diagnosis of HCM was based on the presence of a hypertrophied left ventricle (≥15 mm) in the absence of other diseases capable of producing that magnitude of hypertrophy^2^. Echocardiographic parameters were measured as previously described^29^.

Patients with one or more of the following conditions were excluded: other congenital syndromes (e.g., Noonan’s Syndrome), a history of invasive procedures to treat LVOTO (ASA, surgical myectomy, or dual chamber pacing).

### Classification of patients

Patients were classified into 2 groups based on the therapeutic procedures: 1) ASA group, comprising patients who underwent (at any point during the follow-up period) ASA for management of LVOTO; 2) the control group, comprising patients of NOHCM.

### Invasive procedures

All patients who underwent ASA to treat LVOTO were complicated with severe drug-refractory limiting symptoms [defined as New York Heart Association (NYHA) functional class III/IV dyspnea, Canadian Cardiac Society (CCS) class III/IV angina, or recurrent syncope despite optimal medical therapy or being unable to tolerate medical therapy] and a peak left ventricular outflow tract pressure gradient (LVOTPG) ≥ 50 mm Hg at rest or with provocation, and absence of need for surgical correction of mitral valve or coronary artery disease. Patients who were eligible for both ASA and myocardial myectomy were informed about the risks and benefits of both therapies and were offered the choice between these procedures. ASA were performed as previously reported^30^. Acute technical success of ASA was defined as a decrease in the LVOTPG by at least 50% from baseline immediately after the procedure^13, 31^.

### Follow-up and definition of endpoint

The study endpoint was all-cause mortality and cardiovascular death. The following conditions were considered to be cardiovascular death^32^: 1) procedural mortality of ASA (deaths within 30 days after these procedures); 2) sudden cardiac death (SCD, unexpected within 1 h of witnessed collapse or nocturnal in previously stable patients); 3) heart failure-related death (in the context of progressive cardiac decompensation); 4) stroke-related death (defined according to standard criteria^33^); 5) fatal myocardial infarction (MI). Deaths that occurred within 30 days after an invasive procedure were also counted as SCD, heart failure-related death, stroke-related death, or fatal MI if the corresponding criteria were met.

Follow-up of patients in the NOHCM group began at their first admission since 2000. For patients underwent ASA, follow-up started on the day of operation. In patients who presented LVOTPG ≥50 mm Hg only after provocation, follow-up gradients were assessed with the same provocation. Patients with a resting gradient of ≥50 mm Hg did not undergo provocation before the procedure or in follow-up. The endpoint status was ascertained by follow-up evaluation, including medical records, clinic visits, mailed questionnaires, and telephone contact. For deceased patients, death certificates were procured, and the next of kin were interviewed to determine the cause and time of death. This investigation was according to the principles of the Declaration of Helsinki and approved by the Research Ethics Board of the Fuwai Hospital. Informed consent was obtained from all participants.

### Statistical analysis

Continuous variables were expressed as the mean (standard deviation, SD) or median (interquartile range, IQR), and differences among groups were analyzed using Student’s *t*-test, the Mann-Whitney U test. Categorical variables were summarized as frequencies with percentages and were compared using chi-square test, Fisher’s exact test or the continuity correction chi-square test, where appropriate. Regression analyses of categorical variables were performed by logistic regression analyses. Univariate and multivariable Cox proportional hazards models were used to assess the predictors of the study endpoint. Variables with univariate p values < 0.10 were selected for multivariate analysis and were expressed as hazard ratios (HRs) with 95% confidence intervals (CIs). The following variables were evaluated: age, gender, hypertension, diabetes mellitus, coronary artery disease, atrial fibrillation, prior stroke/TIA, syncope/presyncope, left atrial diameter, left ventricular diameter, ventricular septal thickness, baseline peak pressure gradient, baseline ejection fraction, dosage of alcohol and residual peak pressure gradient. Kaplan-Meier survival curves were constructed to graphically represent survival in each group. Differences in survival were compared with the log-rank test. All analyses were performed with SPSS statistical software, version 22.0 (SPSS Inc.). All tests were 3-tailed, and significance was defined as p < 0.050.

**Table 1:** Baseline Characteristics.

	ASA	NOHCM	p value
	(n = 233)	(n = 297)	
Age, y (SD)	48.7 (9.8)	46.2 (13.6)	0.018
Male, n (%)	149 (63.9)	210 (70.7)	0.112
NYHA class III/IV n (%)	183 (78.5)	11 (3.70)	<0.001
CCS class III/IV n (%)	79 (33.9)	11 (3.70)	<0.001
Syncope/presyncope n (%)	113 (48.5)	27 (9.4)	<0.001
Atrial fibrillation, n (%)	20 (8.6)	50 (16.8)	0.009
Prior stroke/TIA, n (%)	6 (2.6)	9 (3.0)	0.798
Diabetes mellitus, n (%)	11 (4.7)	21 (7.3)	0.272
Hypertension, n (%)	67 (28.8)	91 (31.7)	0.503
Coronary artery disease, n (%)	21 (9.0)	34 (11.9)	0.318
LAD, mm (SD)	41.3 (6.1)	39.6 (7.0)	0.002
IVS, mm (SD)	20.8 (4.9)	21.0 (6.6)	0.740
LVEDd, mm (SD)	41.8 (5.2)	44.7 (5.7)	<0.001
LVEF, % (SD)	71.6 (7.9)	65.4 (8.2)	<0.001
LVOTPG, median (IQR), mm Hg	93.5 (38.1)		<0.001
Baseline medications n (%)			
Beta-blocker	193 (82.8)	258 (86.9)	0.220
CCB	64 (27.5)	70 (23.6)	0.316

ASA, alcohol septal ablation; NOHCM, non obstructive hypertrophic cardiomyopathy; NYHA, New York Heart Association; CCS, Canadian Cardiovascular Society; LAD, left atrial diameter; IVS, intra-ventricular septal thickness; LVEDd, left ventricular end-diastolic diameter; LVEF, left ventricular ejection fraction; LVOTPG, left ventricular outflow tract pressure gradient; SD, standard deviation; CCB, calcium channel blocker.

### Data availability statement

The datasets generated during and/or analysed during the current study are available from the corresponding author on reasonable request.
